# Identification of differentially expressed circular RNAs in human monocyte derived macrophages response to *Mycobacterium tuberculosis* infection

**DOI:** 10.1038/s41598-017-13885-0

**Published:** 2017-10-20

**Authors:** Zikun Huang, Rigu Su, Zhen Deng, Jianqing Xu, Yiping Peng, Qing Luo, Junming Li

**Affiliations:** 1 Department of Clinical Laboratory, the First Affiliated Hospital of Nanchang University, Nanchang, 330006 China; 2 Department of Tuberculosis, Jiangxi Chest Hospital, Nanchang, 330006 China

## Abstract

Macrophages act as the first line of host immune defense against *Mycobacterium tuberculosis* (Mtb). Recent studies have demonstrated circular RNAs (circRNAs) are implicated in a variety of physiological and pathological processes; however, the role of circRNAs in macrophages response to Mtb infection remain unknown. To address this issue, here we characterized circRNAs expression profiles in human monocyte derived macrophages (MDMs) response to Mtb infection using microarray assay. Our results revealed that many circRNAs were differentially expressed in human MDMs after Mtb infection; of these, 32 circRNAs were up-regulated and 110 were down-regulated. Real time PCR results were generally consistent with the microarray data. Furthermore, we found that hsa_circ_0043497 and hsa_circ_0001204 may be effective diagnostic biomarkers for TB. This study provides the first evidence that circRNAs alterations are involved in human MDMs response to TB infection and reveal potential targets for diagnostics and the treatment of TB.

## Introduction

Tuberculosis (TB), an infectious disease caused by *Mycobacterium tuberculosis* (Mtb) infection, remains a leading cause of morbidity and mortality worldwide^1^. In 2015, World Health Organization (WHO) reported that an estimated 10.3 million cases of TB occurred and that 1.4 million died of TB^2^. Despite the high rate of Mtb infection in humans, especially in developing countries, only 5–10% of infected people develop active TB in their lifetime^3^. Interactions between Mtb and the host largely determine the development and outcome of TB infection.

Macrophages play a critical role in the host immune response against mycobacterial infection^4^. They serve as the major host cell niche for intracellular growth and persistence of Mtb during all phases of TB, from primary infection with bacillary dissemination, through latency and reactivation TB^5^. Despite several studies that have been conducted on Mtb-macrophages interaction, however, the underlying molecular regulation is not fully understood.

Circular RNAs (circRNAs) are a special type of non-coding RNA that are formed from the covalent linkage of the 3′ and 5′ ends to form a closed loop^6^. Recent evidence has suggested that circRNAs play essential roles in various physiological and pathological processes^7,8^. Many circRNAs are abundant, stable, conserved and potentially function as competing endogenous RNAs^9^. Due to a lack of 3′ and 5′ ends and resistance to RNases, circRNAs might be used as potential biomarkers and treatment targets for human diseases^10^. With the increasing studies about circRNAs, researchers have reported that circRNAs are involved in the development of several types of diseases, such as cancer^11^, cardiovascular disease^12^, and neurological disorders^13^. However, the role of circRNAs within macrophages innative response to TB infection has yet to be explored.

In this study, we analysed the expression patterns of circRNAs in Mtb-infected human monocyte derived macrophages (MDMs) using microarray assay. Our data revealed that a number of circRNAs were consistently altered under Mtb infection. And then, we demonstrated that hsa_circ_0043497 and hsa_circ_0001204 may be effective diagnostic biomarkers for TB. These findings provided novel insight into the pathogenesis of TB and provide a basis for the diagnosis and therapy of TB.

## Materials and Methods

### Ethics Statement

This study was approved by the ethical committee of the First Affiliated Hospital of Nanchang University and conducted in accordance with the Declaration of Helsinki. All participants provided informed consent before commencement of the study.

### Sample collection

The peripheral blood samples (5 mL) used to validate candidate biomarkers were collected from 96 patients with active pulmonary TB and 85 healthy controls. Patients with pulmonary TB were recruited from the Jiangxi Chest Hospital and the First Affiliated Hospital of Nanchang University from January 2016 to July 2017. All of these patients were diagnosed with TB on the basis of the typical TB clinical symptoms, bacterial culture, and imaging examinations, and in accordance with the Health criteria in People’s Republic of China: The legal diagnostic criteria of infectious diseases (WS288–2008) and the revised international definitions in TB control of the WHO. Individuals with malignant tumor, HIV infections were excluded. Subsequently 12 active pulmonary TB (TB naive) inpatients were treated according to prevailing China National Tuberculosis Program guidelines (2HRZE/6HE). peripheral blood samples were obtained at diagnosis prior to treatment initiation and after completion of treatment (six months). After sample collection, peripheral blood mononuclear cells (PBMCs) were freshly isolated by density gradient centrifugation on Ficoll-Paque (Sigma, USA) according to the manufacturer’s protocol. Then, the PBMCs samples were lysed with TRIzol reagent (Invitrogen, USA) and stored at −80 °C.

### Cell purification and culture

The peripheral blood samples (5 mL) were collected from 22 healthy donors. Individuals with cancer, allergic diseases, immune-compromised conditions, diabetes or other infectious diseases such as HBV, HCV and HIV infection were excluded. After sample collection, PBMCs were isolated by density gradient centrifugation. To develop MDMs, the monocytes were purified using anti-CD14 magnetic beads (Miltenyi Biotec, USA) and the purified monocytes were cultured in RPMI 1640 medium contains 10% Human serum and 0.05% Glutamine (Sigma, USA) for 7 days at 5% CO_2_ and 37 °C^14^. The MDMs were identified by morphologic observation and flow cytometric analysis followed by anti-CD68 staining.

### Bacterial strains and culture

Mtb H37Rv strain 27294 was purchased from the American Type Culture Collection (ATCC). They were grown in Middlebrook 7H9 broth containing 10% oleic acid-albumin-dextrose-catalase (OADC) and 0.05% Tween-80 at 37 °C. They were washed with PBS and passed through a syringe, to disrupt the clumps, before infecting human MDMs.

### Cell infection

The human MDMs were plated in 12-well plates, and incubated overnight. The cells were infected at a multiplicity of infection (MOI) of 5. Uninfected cells which received only PBS served as controls. After 4–6 h incubation at 37 °C, non-phagocytosed bacteria were washed off using PBS. MDMs were replenished with fresh RPMI 1640 and incubated for 24 h at 37 °C with 5% CO_2_. After 24 h, cells were harvested and RNA was isolated.

### RNA isolation

Total RNA was extracted using TRIzol reagent (Invitrogen, USA) according to the manufacturer’s protocol. The integrity of the RNA was assessed by electrophoresis on a denaturing agarose gel. A NanoDrop ND-1000 spectrophotometer was used for the accurate measurement of RNA concentration.

### CircRNAs microarray and computational analysis

Human CircRNA Array V2.0 (8 × 15 K) is manufactured by Arraystar Technologies (Rockville, MD, USA). Six MDMs samples, including three infected samples and three uninfected samples, were sent to KangChen Bio-tech (Shanghai, China) for the Arraystar circRNA microarray analysis. Microarray hybridization were performed according to the protocols of Arraystar. The scatter plot is a visualization method used for assessing the circRNA expression variation. Differentially expressed circRNAs with statistical significance (fold changes >2.0 and *P* < 0.05) between groups were identified using fold change cut-off or volcano plot filtering, respectively. The circRNAs/microRNAs (miRNAs) interaction was predicted using Arraystar’s home-made miRNA target prediction software based on TargetScan and miRanda.

### Quantitative real-time PCR

Total RNA (2 μg) was reversely transcribed into cDNA using the Reverse Transcription System Kit (Takara, Dalian, China). The expression levels of circRNAs and miRNAs were determined by quantitative real-time PCR (RT-qPCR) using SYBR Master Mix (Applied Biosystems, Foster City, CA, USA) and mirVanaTM RT-qPCR miRNA Detection Kit (Ambion, Austin, USA) on Applied Biosystems 7500 Real-Time PCR System (Applied Biosystems, Foster City, USA), respectively. Primers used in this study were listed in Supplementary Table 1. The PCR primers of U6 and miRNAs were purchased from RIBOBIO. All RT-qPCR experiments were performed in triplicate. The relative expression levels of circRNAs and miRNAs were calculated by the 2^−ΔΔCt^ method. The the relative expression levels of circRNAs were normalized to GAPDH and the relative expression levels of miRNAs were normalized to U6^10^. PCR product was examined by agarose gel electrophoresis using 2% (w/v) LE agarose (Seakem) stained with ethidium bromide.

### Statistics

Numerical data were shown as the mean ± standard error of the mean (SEM). A one-way ANOVA test, Mann-Whitney test or Student t-test was used for statistical analysis. Receiver operating characteristic (ROC) analysis was used to evaluate the power of candidate circRNAs. All statistical tests were performed with GraphPad Prism 5.0 (GraphPad Software, San Diego, USA). *P* < 0.05 was considered statistically significant.

## Results

### Overview of circRNA profiles

**Figure 1:**
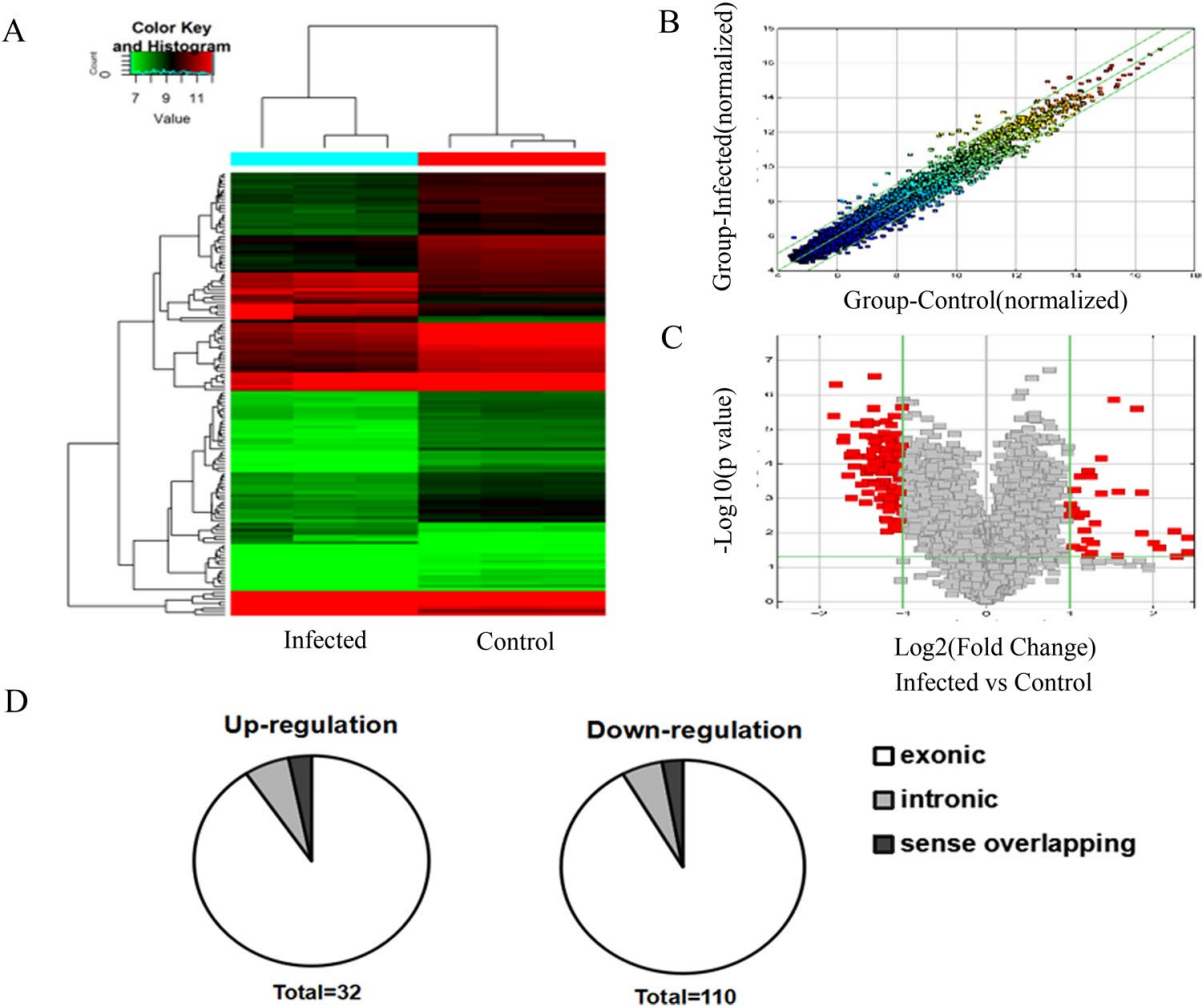
Analysis of differentially expressed circRNAs in Mtb-infected human MDMs and uninfected MDMs. (**A**) Hierarchical clustering results of circRNAs expression profiles among the Mtb-infected group and control group. “Red” indicated high relative expression and “green” indicated low relative expression. (**B**) CircRNAs in the Scatter-Plot above the top green line and below the bottom green line indicated more than 2.0 fold change of circRNAs between the two groups. (**C**) Volcano-Plot of circRNAs expression profile. The vertical lines correspond to 2.0-fold up and down and the horizontal line represents a *P*-value of 0.05. The red point in the plot represents the differentially espressed circRNAs with statistical significance. (**D**) classification of dysregulated circRNAs.

To explore the potential circRNAs involved in human MDMs response to TB infection, we examined the circRNAs expression profiles through microarray analysis. In total, 142 circRNAs were identified with differential expression between Mtb-infected group and uninfected group (fold change >2.0, *P* < 0.05), among which 32 circRNAs were up-regulated while 110 circRNAs were down-regulated (Fig. 1 and Supplementary Table 2). Specifically, the most up-regulated circRNAs were: hsa_circ_0003528, hsa_circ_0001417, hsa_circ_0043497 and hsa_circ_0038929, of which hsa_circ_0003528 was the highest. The most highly down-regulated were: hsa_circ_0068784, hsa_circ_0057090, hsa_circ_0056247 and hsa_circ_0001204, of which hsa_circ_0068784 showed the largest down-regulation. In present study, the top 40 up- and down-regulated circRNAs are listed in Table 1 by fold change. We summarized the classification of dysregulated circRNAs (Fig. 1). Among the up-regulated circRNAs, there were 29 exonic, 2 intronic and 1 sense overlapping. Among the down-regulated circRNAs, there were 101 exonic, 6 intronic and 3 sense overlapping.

**Table 1:** Top 40 differently expressed circRNAs in *M. tuberculosis*-infected human monocyte derived macrophages.

CircRNA ID	*P*-value	FDR	FC	Regulation	CircRNA_type	Chrom	Strand	Best_transcript	GeneSymbol
hsa_circ_0003528	0.037466755	0.115427239	5.341549	up	exonic	chr5	+	NM_021982	SEC. 24 A
hsa_circ_0001417	0.013874737	0.062154272	5.3239089	up	exonic	chr4	—	NM_032217	ANKRD17
hsa_circ_0043497	0.049072061	0.137777454	4.866483	up	exonic	chr17	—	NM_014815	MED24
hsa_circ_0038929	0.008851434	0.04811339	4.7593614	up	exonic	chr16	+	NM_007317	KIF22
hsa_circ_0000994	0.02769979	0.094239935	4.2102057	up	exonic	chr2	—	NM_021097	SLC8A1
hsa_circ_0001216	0.019989045	0.076864421	4.0183976	up	sense overlapping	chr22	—	NM_005080	XBP1
hsa_circ_0007705	0.000698384	0.012309287	3.6367029	up	exonic	chr15	+	NM_016396	CTDSPL2
hsa_circ_0030045	0.010558813	0.053165131	3.6256505	up	exonic	chr13	+	NM_014252	SLC25A15
hsa_circ_0006682	2.5579E-06	0.002191928	3.4860902	up	intronic	chr17	+	ENST00000439730	GOSR2
hsa_circ_0005108	0.000648282	0.011824622	2.9961246	up	exonic	chr14	—	NM_203301	FBXO33
hsa_circ_0028152	0.046309846	0.132935223	2.9723266	up	exonic	chr12	—	NM_014776	GIT2
hsa_circ_0003748	1.35271E-06	0.002016717	2.8810825	up	exonic	chr3	—	NM_016291	IP6K2
hsa_circ_0076260	6.90949E-05	0.005050619	2.5996407	up	exonic	chr6	+	NM_001206927	DNAH8
hsa_circ_0083157	0.000732236	0.012499195	2.5949787	up	exonic	chr7	+	NM_014671	UBE3C
hsa_circ_0008157	0.005409066	0.035950336	2.4669575	up	exonic	chr11	—	NM_020153	IFT46
hsa_circ_0007209	0.020193068	0.077300669	2.4254376	up	exonic	chr8	—	NM_020863	ZFAT
hsa_circ_0002824	0.037701504	0.115881588	2.392587	up	exonic	chr9	+	NM_004972	JAK2
hsa_circ_0022382	0.000235706	0.007462334	2.376217	up	exonic	chr11	+	NM_004265	FADS2
hsa_circ_0091994	0.000162781	0.006608264	2.3325785	up	exonic	chrX	—	NM_001456	FLNA
hsa_circ_0026616	0.047782317	0.135397186	2.3247835	up	exonic	chr12	—	NM_015665	AAAS
hsa_circ_0068784	4.17569E-06	0.002492284	3.5480508	down	exonic	chr4	+	NM_003441	ZNF141
hsa_circ_0057090	4.9948E-07	0.00147459	3.4927401	down	exonic	chr2	+	NM_002610	PDK1
hsa_circ_0056247	2.22076E-05	0.003841024	3.2888045	down	exonic	chr2	+	NM_002830	PTPN4
hsa_circ_0001204	1.64596E-05	0.003817991	3.2644161	down	intronic	chr22	+	ENST00000351989	DGCR8
hsa_circ_0001747	0.000218714	0.007377021	3.149758	down	exonic	chr7	+	NM_013255	MKLN1
hsa_circ_0017311	5.30031E-05	0.004765914	3.0933966	down	exonic	chr1	+	NM_152609	CNST
hsa_circ_0047744	5.99584E-05	0.004765914	3.0829551	down	exonic	chr18	+	NM_015285	WDR7
hsa_circ_0030569	0.001000539	0.014333503	3.0627677	down	exonic	chr13	—	NM_005845	ABCC4
hsa_circ_0089866	4.7015E-05	0.004765914	3.0233465	down	exonic	chrX	+	NM_015691	WWC3
hsa_circ_0023748	0.000120414	0.006050795	2.9884338	down	exonic	chr11	—	NM_033547	INTS4
hsa_circ_0008153	6.89986E-06	0.002809671	2.92489	down	exonic	chr15	+	NM_006537	USP3
hsa_circ_0004893	6.21515E-05	0.004860577	2.9098745	down	exonic	chr1	—	NM_080391	PTP4A2
hsa_circ_0012425	0.000394458	0.009352714	2.8215173	down	exonic	chr1	—	NM_001981	EPS15
hsa_circ_0039261	1.57454E-05	0.003817991	2.7291055	down	exonic	chr16	+	NM_000293	PHKB
hsa_circ_0037680	0.001355771	0.016823871	2.72776	down	exonic	chr16	—	NM_004380	CREBBP
hsa_circ_0000028	7.56027E-06	0.002879975	2.696051	down	exonic	chr1	—	NM_032236	USP48
hsa_circ_0008205	0.000125451	0.00607151	2.6958097	down	exonic	chr8	—	NM_144649	TMEM71
hsa_circ_0003615	6.03401E-06	0.002727301	2.6943212	down	exonic	chr11	—	NM_024079	ALG8
hsa_circ_0002383	0.000178628	0.006792563	2.6911006	down	exonic	chr11	—	NM_006645	STARD10
hsa_circ_0003768	0.000673145	0.0120992	2.6750548	down	exonic	chr11	+	NM_003477	PDHX

Note: CircRNA ID: The circRNA ID is in circBase (http://www.circbase.org/). *P*-value: *P*-value calculated from paired t-test. FDR: FDR is calculated from Benjamini Hochberg FDR. Fold Change: Te absolute ratio (no log scale) of normalized intensities between two conditions.

### Validation of circRNAs expression

**Figure 2:**
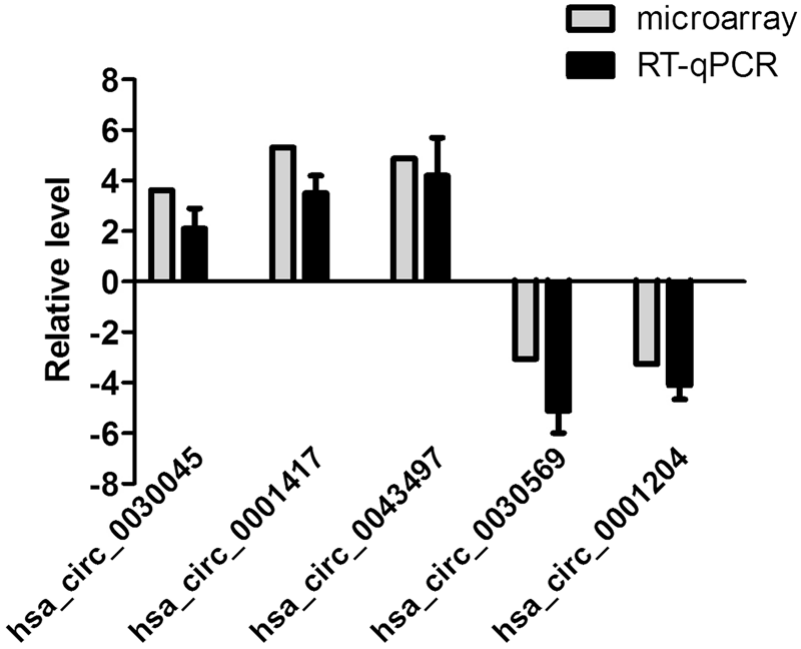
Confirmation of the differential expression of circRNAs by RT-qPCR. Five differentially expressed circRNAs were validated by RT-qPCR. Individual RT-qPCR assays were performed using samples from six additional human MDMs after 24 h of infection with H37Rv. Data are expressed as the means ± SEM. The relative expression levels of circRNAs were normalized to levels of GAPDH.

To validate microarray analysis findings, we randomly selected 5 circRNAs from the differentially expressed circRNAs with fold change >3.0 and analyzed their expression by RT-qPCR in expanded MDMs samples. The results showed that these circRNAs were significantly differentially expressed. Particularly, hsa_circ_0030045, hsa_circ_0001417 and hsa_circ_0043497 are significantly up-regulated in Mtb-infected group, while hsa_circ_0030569 and hsa_circ_0001204 are significantly down-regulated in Mtb-infected group (Fig. 2). Our data confirmed the findings of the microarray analysis.

### Diagnostic value of hsa_circ_0043497 and hsa_circ_0001204 expression levels in TB

**Figure 3:**
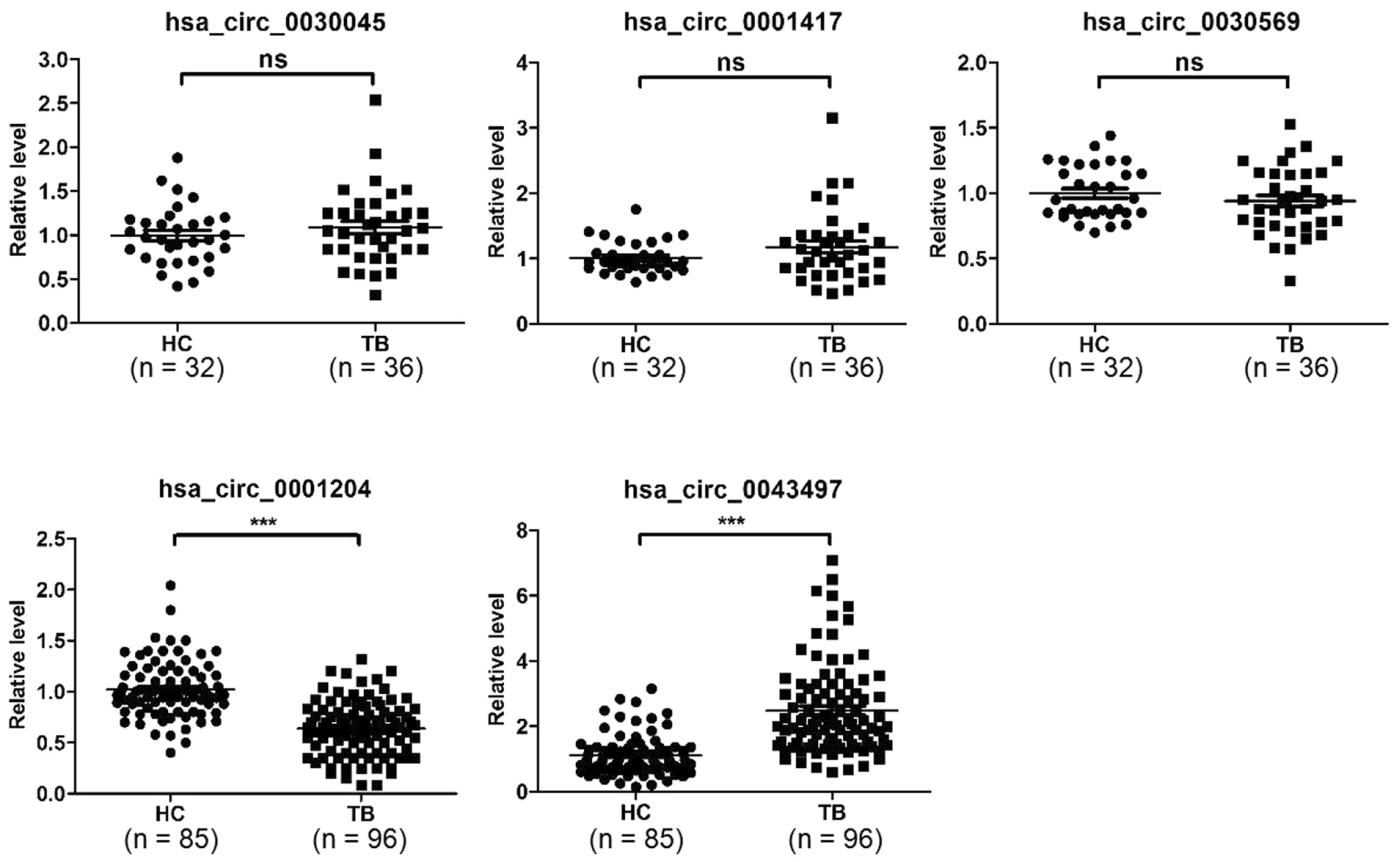
Comparison of circRNAs levels in active TB patients and healthy control. The expression of five circRNAs in 96 pulmonary TB patients and 85 healthy controls were measured by RT-qPCR. Statistical analysis was performed using the nonparametric Mann-Whitney test. The relative expression levels of circRNAs were normalized to levels of GAPDH. ***P < 0.001.

**Figure 4:**
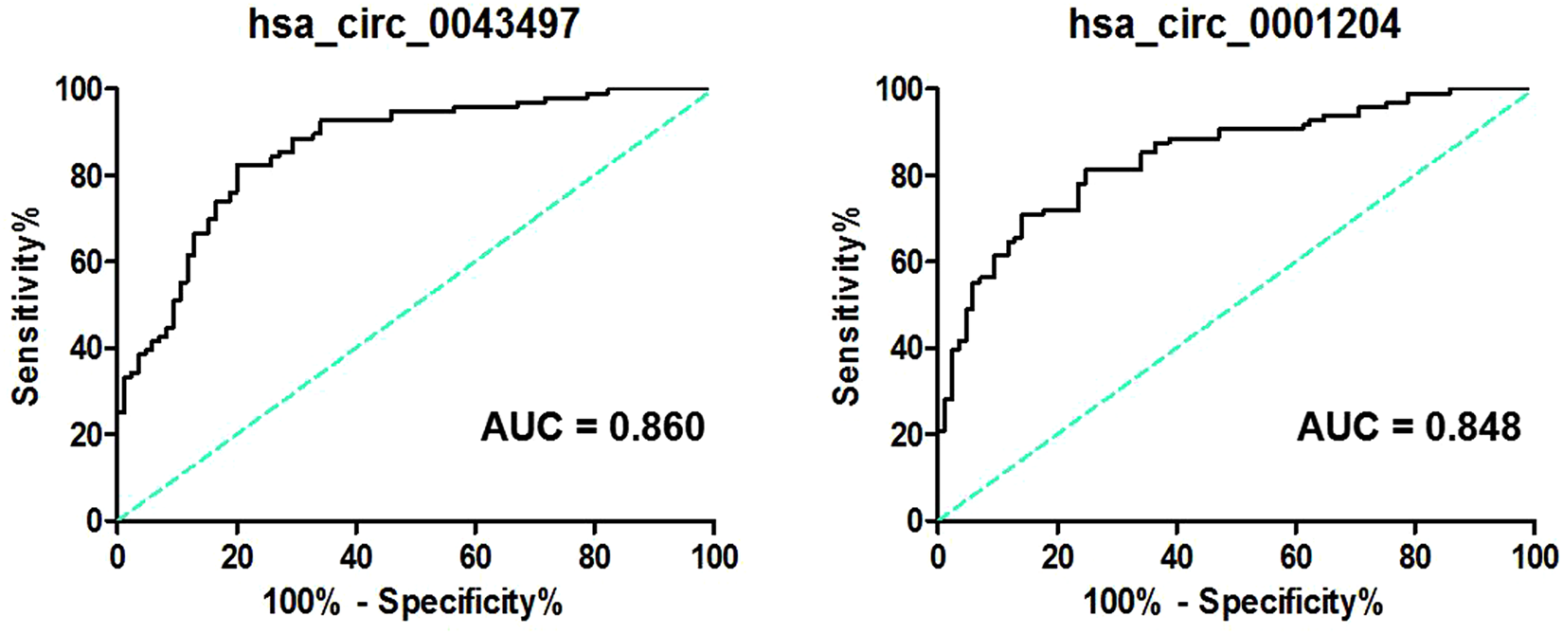
Receiver operating characteristic (ROC) curves of hsa_circ_0043497 and hsa_circ_0001204 between active TB patients and healthy control. ROC curves of hsa_circ_0043497 and hsa_circ_0001204 showed a moderate distinguishing efficiency.

Recently, many studies have demonstrated that circRNAs can serve as novel diagnostic markers for diseases. Thus, we sought to determine whether the circRNAs identified in our microarray analysis could potentially serve as diagnostic biomarkers for TB. The levels of 5 circRNAs (hsa_circ_0030045, hsa_circ_0001417, hsa_circ_0043497, hsa_circ_0030569 and hsa_circ_0001204) were measured in PBMCs samples from 96 patients with active pulmonary TB and 85 healthy controls using RT-qPCR. The demographic characteristics of the participants are showed in Table 2. Of the five circRNAs studied, the levels of hsa_circ_0043497 were significantly elevated and hsa_circ_0001204 were significantly reduced in PBMCs of TB patients as compared to healthy controls. There were no significant differences in the level of hsa_circ_0030045, hsa_circ_0001417 and hsa_circ_0030569 between TB patients and controls (Fig. 3). To compare the diagnostic value of hsa_circ_0043497 and hsa_circ_0001204 as candidate biomarkers of TB, we performed ROC curve analysis for hsa_circ_0043497 and hsa_circ_0001204. ROC curve analysis exhibited a significant distinguishing efficiency with an area under the curve (AUC) value of 0.860 for hsa_circ_0043497 and 0.848 for hsa_circ_0001204 (Fig. 4). These results suggest that altered expression of hsa_circ_0043497 and hsa_circ_0001204 in PBMCs are potential novel biomarkers for the diagnosis of TB.

**Table 2:** Demographic and clinical characteristics of TB patients and healthy controls.

	Active TB patients (n = 96)	Healthy controls (n = 85)
Sex (female/male)	96 (40/56)	85 (33/52)
Age (mean ± SD)	34.57 ± 4.83	36.19 ± 5.06
ELISPOT-positive	96/96	0/85
*M. tuberculosis* culture-positive	58/96	N/A
Lung cavity	52/96	0/85
New/retreatment	80/16	N/A

Note: N/A: not applicable.

### Effects of anti-TB therapy on level of hsa_circ_0043497 and hsa_circ_0001204

**Figure 5:**
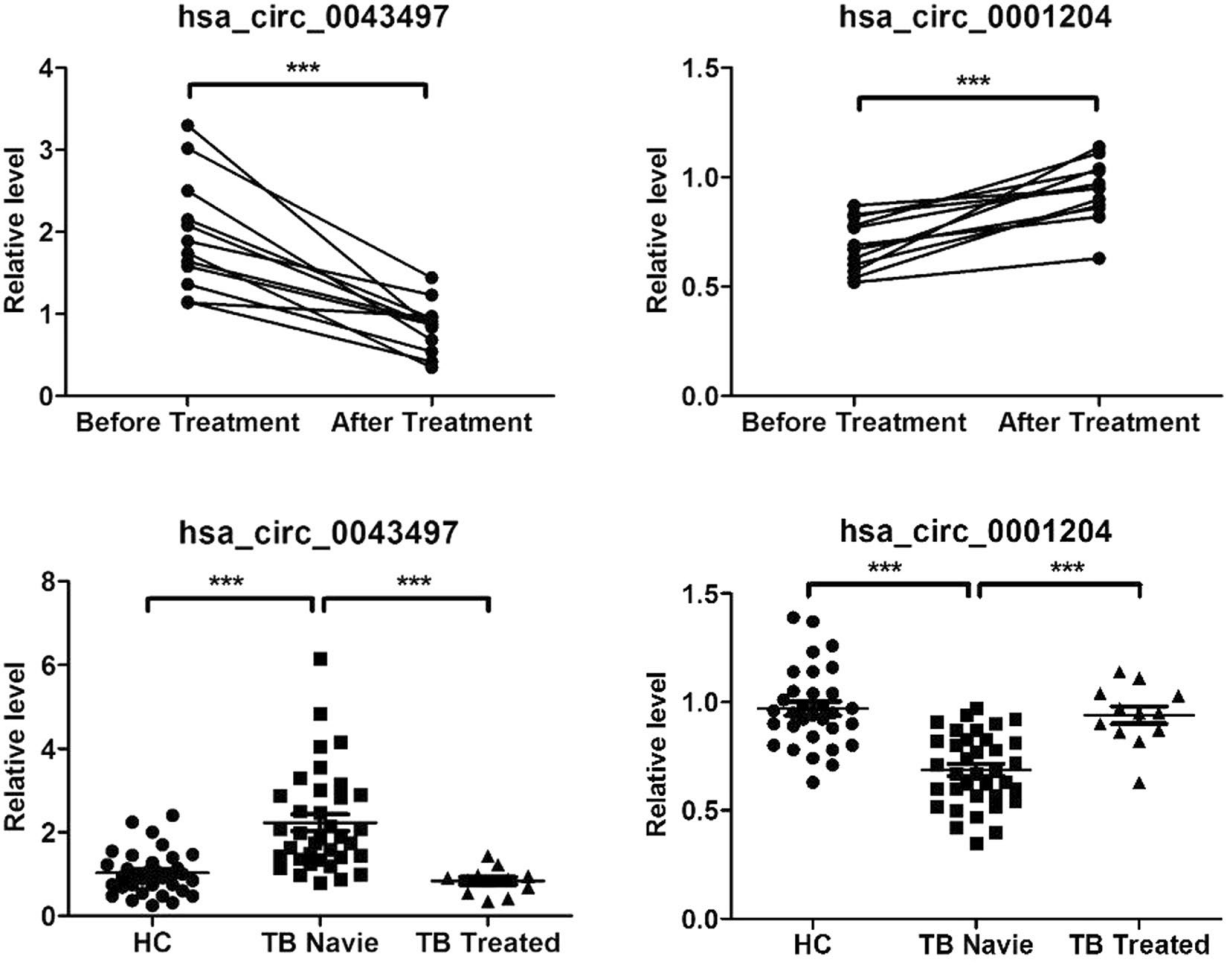
Change in the levels of hsa_circ_0043497 and hsa_circ_0001204 in response to therapy. The levels of hsa_circ_0043497 and hsa_circ_0001204 were measured by RT-qPCR. Dot plot shows relative levels of hsa_circ_0043497 and hsa_circ_0001204 in the same patients before and after completion of therapy (n = 12). Relative levels of hsa_circ_0043497 and hsa_circ_0001204 in healthy controls (n = 32), pulmonary TB naive patients (n = 36) and TB treated group (n = 12). The value between the healthy control and TB treated group is not significantly different. ***P < 0.001.

Furthermore, we compared the expression of hsa_circ_0043497 and hsa_circ_0001204 in 12 patients before and after TB therapy. As compared to their pre-treatment, the levels of hsa_circ_0043497 decreased after anti-TB treatment. As compared to controls, the mean levels of hsa_circ_0043497 which was higher in TB infected group came to near normal after therapy. The mean hsa_circ_0043497 levels did not differ significantly between healthy control and TB treated group. As compared to healthy controls, the mean levels of hsa_circ_0001204 were significantly lower in TB infected group; the levels increase post therapy. The mean hsa_circ_0001204 levels did not differ significantly between healthy control and TB treated group (Fig. 5).

### Target gene prediction

**Figure 6:**
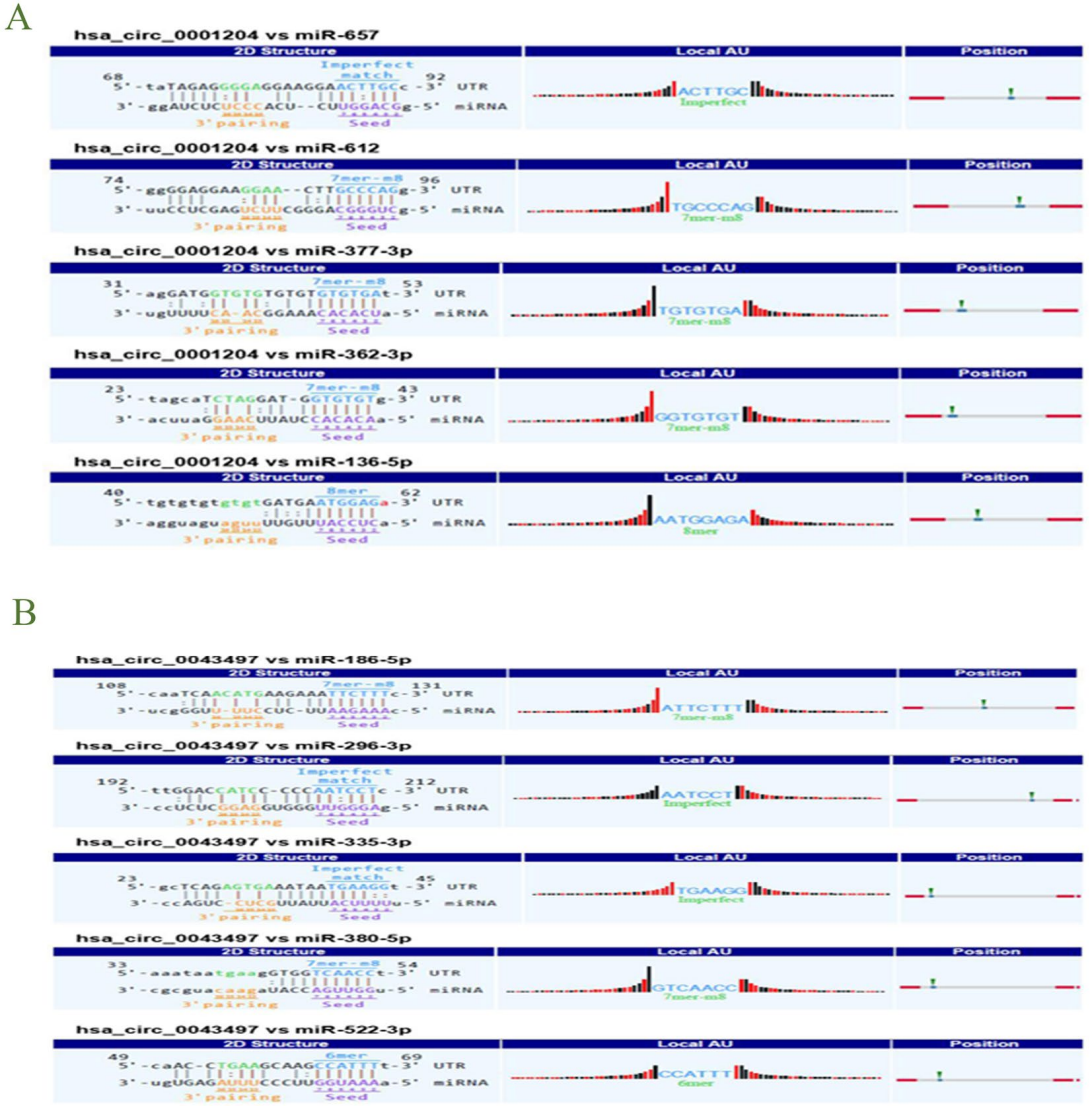
A snippet of the detailed annotation for circRNAs/miRNAs interaction. (**A**) hsa_circ_0001204. (**B**) hsa_circ_0043497. The circRNAs/miRNAs interaction was predicted with Arraystar’s home-made miRNA target prediction software based on TargetScan and miRanda. Binding sites of conserved miRNAs with good mirSVR scores are represented.

**Figure 7:**
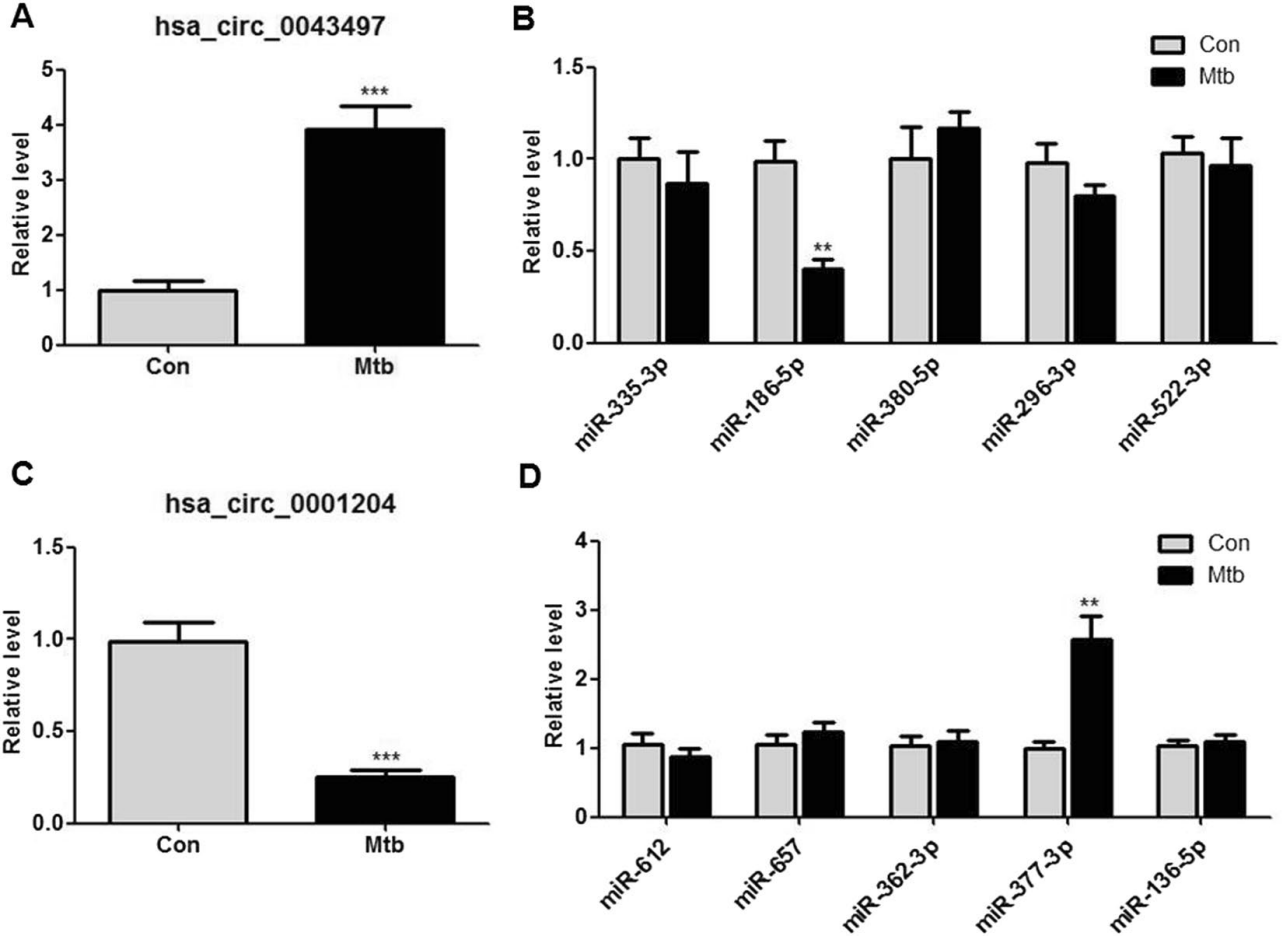
Expression levels of hsa_circ_0043497 and hsa_circ_0001204 and their target miRNAs in Mtb-infected human MDMs. RT-qPCR was performed to evaluate the expression levels of hsa_circ_0043497 (**A**) and its top 5 predicted miRNA targets (miR-335-3p, miR-186-5p, miR-380-5p, miR-296-3p and miR-522-3p) (**B**), hsa_circ_0001204 (**C**) and its top 5 predicted miRNA targets (miR-612, miR-657, miR-362-3p, miR-377-3p and miR-136-5p) (**D**) in ten human MDMs after 24 h of infection with H37Rv. Data are expressed as the means ± SEM. The relative expression levels of circRNAs were normalized to levels of GAPDH and the relative expression levels of miRNAs were normalized to levels of U6. **P < 0.01, ***P < 0.001 compared to the control group.

To evaluate circRNAs potential functions, we investigated potential miRNAs binding with circRNAs using Arraystar’s home-made miRNA target prediction software. We selected 18 differentially expressed circRNAs with the highest fold-change (fold-change > 3.0; *P* < 0.05) to predict their miRNA response elements (MREs), including 9 up-regulated circRNAs and 9 down-regulated circRNAs. Five MREs with good mirSVR scores for each circRNA are shown in Table 3. In the study, two confirmed circRNAs (hsa_circ_0043497 and hsa_circ_0001204) and their target miRNAs were focused (Fig. 6). Then, the expression levels of hsa_circ_0043497-target miRNAs and hsa_circ_0001204-target miRNAs were validated by RT-qPCR in ten additional human MDMs after 24 h of infection with Mtb. Of the ten miRNAs studied, the levels of miR-186–5p were significantly reduced and the levels of miR-377-3p were significantly elevated in Mtb-infected MDMs (Fig. 7).

**Table 3:** Annotation for differentially expressed circRNAs/miRNAs interaction.

CircRNA ID	*P*-value	FDR	FC	Regulation	MRE1	MRE2	MRE3	MRE4	MRE5
hsa_circ_0003528	0.037466755	0.115427239	5.341549	up	miR-421	miR-181a-2-3p	miR-505-3p	miR-429	miR-380-5p
hsa_circ_0001417	0.013874737	0.062154272	5.3239089	up	miR-298	miR-766-5p	miR-323a-5p	miR-138-5p	miR-486-3p
hsa_circ_0043497	0.049072061	0.137777454	4.866483	up	miR-335-3p	miR-186-5p	miR-380-5p	miR-296-3p	miR-522-3p
hsa_circ_0038929	0.008851434	0.04811339	4.7593614	up	miR-485-5p	miR-221-5p	miR-492	miR-182-5p	miR-545-3p
hsa_circ_0000994	0.02769979	0.094239935	4.2102057	up	miR-27b-3p	miR-27a-3p	miR-373-5p	miR-335-3p	miR-628-5p
hsa_circ_0001216	0.019989045	0.076864421	4.0183976	up	miR-326	miR-3154	miR-4747-5p	miR-676-5p	miR-330-5p
hsa_circ_0007705	0.000698384	0.012309287	3.6367029	up	miR-103a-2-5p	miR-190a-5p	miR-578	miR-22-5p	miR-190b
hsa_circ_0030045	0.010558813	0.053165131	3.6256505	up	miR-650	miR-605-3p	miR-876-5p	miR-526b-5p	miR-569
hsa_circ_0006682	2.5579E-06	0.002191928	3.4860902	up	miR-485-5p	miR-6884-5p	miR-377-5p	miR-6848-5p	miR-6866-5p
hsa_circ_0068784	4.17569E-06	0.002492284	3.5480508	down	miR-4673	miR-3692-5p	miR-6754-5p	miR-23a-3p	miR-7113-5p
hsa_circ_0057090	4.9948E-07	0.00147459	3.4927401	down	miR-1193	miR-548k	miR-643	miR-3064-5p	miR-4299
hsa_circ_0056247	2.22076E-05	0.003841024	3.2888045	down	miR-450a-2-3p	miR-23b-5p	miR-23a-5p	miR-511-5p	miR-7-5p
hsa_circ_0001204	1.64596E-05	0.003817991	3.2644161	down	miR-612	miR-657	miR-362-3p	miR-377-3p	miR-136-5p
hsa_circ_0001747	0.000218714	0.007377021	3.149758	down	miR-616-5p	miR-30d-3p	miR-320b	miR-320a	miR-302c-5p
hsa_circ_0017311	5.30031E-05	0.004765914	3.0933966	down	miR-767-3p	miR-412-3p	miR-335-3p	miR-191-3p	miR-421
hsa_circ_0047744	5.99584E-05	0.004765914	3.0829551	down	miR-503-5p	miR-424-5p	miR-15a-5p	miR-16-5p	miR-497-5p
hsa_circ_0030569	0.001000539	0.014333503	3.0627677	down	miR-30d-3p	miR-29a-5p	miR-584-3p	miR-30a-3p	miR-30e-3p
hsa_circ_0089866	4.7015E-05	0.004765914	3.0233465	down	miR-1914-5p	miR-556-5p	miR-3692-5p	miR-4268	miR-4640-5p

Note: We selected 18 differentially expressed circRNAs with the highest fold-change (fold-change >3.0; *P* < 0.05) to predict their miRNAs response elements (MREs), including 9 up-regulated circRNAs and 9 down-regulated circRNAs. The circRNAs/miRNAs interaction was predicted with Arraystar’s home-made miRNA target prediction software based on TargetScan and miRanda.

## Discussion

CircRNAs are a new class of long non-coding RNAs which do not have 5′ or 3′ ends but are covalently linked to form a closed circular structure. Recent evidence has suggested that circRNAs play essential roles in various physiological and pathological processes^15^. The dysregulation of circRNAs have also been implicated in the occurrence and progression of many human diseases^16^. However, the relationship between circRNAs and TB infection has not been reported.

In this study, we comprehensively profiled the circRNAs expression of human MDMs response to Mtb infection in order to improve our understanding of the pathogenesis of TB infection as well as identify potential circRNAs biomarkers for TB. We demonstrated that the expression of 142 circRNAs was significantly different in the Mtb-infected group compared with uninfected group. Among these, 32 circRNAs were up-regulated, while 110 circRNAs were down-regulated. Most of differentially expressed circRNAs originate from the exons. Some are from introns, while, a few are other sources. We then randomly selected five circRNAs from the differentially expressed cricRNAs with a fold change >3.0 and performed a quantitative real time PCR to examine these circRNAs’ expression levels in Mtb-infected MDMs. RT-qPCR results were generally consistent with the microarray data. To our knowledge, this is the first report on the expression of circRNAs in the human MDMs response to TB infection.

Early diagnosis of TB infection is essential for controlling the spread of the disease and providing early therapy for TB epidemic^17^. Several groups have reported the potential use of circRNAs as promising biomarkers for detection of human diseases^18,19^. For example, recently, Cui *et al*.^20^ showed that hsa_circRNA_103636 in PBMCs can be used as a novel biomarker for the diagnosis and treatment of major depressive disorder. Chen *et al*.^21^ reported that hsa_circ_0000190 yields a diagnostic characteristic with a sensitivity of 71.2% and a specificity of 75.0% for detection of gastric cancer. However, there are no reports regarding the use of circRNAs as biomarkers for TB. In this study, there were many differentially expressed circRNAs induced by Mtb infection, which indicated that circRNAs have the potential to be novel biomarkers for TB. To identify the clinically applicable biomarkers, 5 significant difierentially expressed circRNAs were selected for further analysis. We found the expression of hsa_circ_0043497 and hsa_circ_0001204 were significantly elevated in TB infected patients compared to healthy subjects. In ROC plots, the AUC was 0.856 for hsa_circ_0043497 and 0.882 for hsa_circ_0001204. Next, we investigated the effect of anti-TB drug treatment on hsa_circ_0043497 and hsa_circ_0001204 expression. In the present study, we observed that the levels of hsa_circ_0043497 and hsa_circ_0001204 which were altered in naive TB patients reverted to normal after completion of anti-TB therapy and proven to be acid-fast bacillius (AFB) negative in sputum. However, a larger sample size is needed to confirm our results.

Some studies have revealed that circRNAs could function as miRNAs sponges, modulate alternative splicing or transcription, and regulate the expression of parental genes^7,8^. To evaluate hsa_circ_0043497 and hsa_circ_0001204 potential functions, we investigated potential miRNAs binding with hsa_circ_0043497 and hsa_circ_0001204. The potential miRNAs targets of hsa_circ_0043497 include miR-335-3p, miR-186-5p, miR-380-5p, miR-296-3p and miR-522-3p. For hsa_circ_0001204, the potential miRNAs targets include miR-612, miR-657, miR-362-3p, miR-377-3p and miR-136-5p. These potential target miRNAs were verified in MDMs with or without Mtb infection using RT-qPCR. And the data of RT-qPCR showed that the expression levels of miR-377-3p were markedly elevated and the expression levels of miR-186-5p were significantly reduced in Mtb-infected MDMs. However, due to the limited known function of circRNAs and miRNAs, a lot of circRNAs/miRNAs interactions should be analyzed in the future.

In conclusion, in the study, we described differentially expressed circRNAs in human MDMs response to TB infection. Furthermore, hsa_circ_0043497 and hsa_circ_0001204 were identified as potential non-invasive molecular markers for rapid diagnosis of TB. Our findings shed a novel light on our understandings of the pathogenesis of TB infection. To our knowledge, this is the first research addressing circRNAs expression profiles in macrophages response to TB infection. Further studies should focus on the function of circRNAs involved in TB infection, which may lead to new theories for TB pathogenesis and give new potentially therapeutic targets in active TB.

## Electronic supplementary material

Table s1

Dataset 1
