# Supplementary material for: Identification of differentially expressed circular RNAs in human monocyte derived macrophages response to Mycobacterium tuberculosis infection
Source: Sci Rep. 2017 Oct 20;7:13673. doi: 10.1038/s41598-017-13885-0 (PMC5651861; doi:10.1038/s41598-017-13885-0)
Supplement: Supplementary file 1 — Table s1 [file 41598_2017_13885_MOESM1_ESM.doc]

# Identification of differentially expressed circular RNAs in human monocyte derived macrophages response to *Mycobacterium tuberculosis* infection

Zikun Huang^1^, Rigu Su^1^, Zhen Deng^1^, Jianqing Xu^1^, Yiping Peng^2^, Qing Luo^1*^, Junming Li^1*^

^1^ Department of Clinical Laboratory, the First Affiliated Hospital of Nanchang University, Nanchang 330006, China.

^2^ Department of Tuberculosis, Jiangxi Chest Hospital, Nanchang 330006, China.

| **Table s1. Primers used for RT-qPCR analysis of circRNAs levels** | | |
| --- | --- | --- |
| **Name** | **Sequences** | |
| GAPDH | Forward | 5'-GCACCGTCAAGGCTGAGAAC-3' |
|  | Reverse | 5'-TGGTGAAGACGCCAGTGGA-3' |
| hsa_circ_0030045 | Forward | 5'-TCCTCTTCATGTGCTACGGC-3' |
|  | Reverse | 5'-TTGTGGAAGGCAGCTCTCTG-3' |
| hsa_circ_0001417 | Forward | 5'-ACCCGCAGCTGCTAACTTT-3' |
|  | Reverse | 5'-TCAATTTCTTTGTCTGGATCCTTG-3' |
| hsa_circ_0043497 | Forward | 5'-TCAACCTGGCAGATGCGTTA-3' |
|  | Reverse | 5'-TTTCACTCTGAGCAGGTGGC-3' |
| hsa_circ_0030569 | Forward | 5'-AAGATGGTGCAACAACTGGG-3' |
|  | Reverse | 5'-TGCGATCCAAGGAATCACGG-3' |
| hsa_circ_0001204 | Forward | 5'-GCTTATAGAGGGGAGGAAGGAAC-3' |
|  | Reverse | 5'-CATCCTAGATGCTACAGACACAA-3' |
